# Biomarkers of dietary PUFA intake in childhood and adolescence in relation to cardiometabolic risk factors in young adulthood: a prospective cohort study in Sweden

**DOI:** 10.1016/j.ajcnut.2024.11.029

**Published:** 2025-02-07

**Authors:** Annachiara Malin Igra, Sandra Ekström, Niklas Andersson, Petter Ljungman, Erik Melén, Inger Kull, Ulf Risérus, Anna Bergström

**Affiliations:** 1 Institute of Environmental Medicine, Karolinska Institutet, Stockholm, Sweden; 2 Center for Occupational and Environmental Medicine, Region Stockholm, Stockholm, Sweden; 3 Department of Clinical Science and Education, Södersjukhuset, Karolinska Institutet, Stockholm, Sweden; 4 Department of Cardiology, Danderyd University Hospital, Danderyd, Sweden; 5 Sachs’ Children and Youth Hospital, Södersjukhuset, Stockholm, Sweden; 6 Clinical Nutrition and Metabolism, Department of Public Health and Caring Sciences, Uppsala University, Uppsala, Sweden

**Keywords:** PUFAs, obesity, cardiometabolic risk factors, longitudinal study, cohort study

## Abstract

**Background:**

PUFAs, especially from vegetable fat sources, have been suggested to contribute to weight regulation and be protective to cardiometabolic health. However, a few longitudinal studies on childhood exposure are available, with short follow-up time and conflicting results.

**Objectives:**

To study the relationship between plasma proportions of PUFA in childhood and adolescence and cardiometabolic risk factors in young adulthood, such as obesity, body composition, blood pressure (BP), and blood lipids in a prospective cohort study.

**Methods:**

We included *n* = 688 participants of the BAMSE (Barn, Allergi, Miljö, Stockholm, Epidemiologi) cohort in Stockholm, Sweden, with data on plasma phospholipid proportions of n-3 and n-6 fatty acids [α-linolenic acid (ALA), EPA, docosapentaenoic acid, DHA, linoleic acid (LA), and arachidonic acid (AA)] at 8 and 16 y and body mass index (BMI), waist circumference, fat mass %, BP, and blood lipids at 24 y. Associations between PUFAs and cardiometabolic health outcomes were assessed with sex-stratified multivariable-adjusted linear and logistic regression models.

**Results:**

In females, LA and ALA at 16 y were inversely associated with BMI [B: −0.35 (−0.54, −0.17) and B: −6.1 (−11, −1.5), respectively], and similarly with waist circumference and fat mass at 24 y. Also in females, LA was inversely associated with BP, triglycerides, LDL-cholesterol), and total cholesterol (e.g., B −0.044 [−0.079, −0.0099] for LA at 16 y and LDL-cholesterol), whereas ALA was only inversely associated with LDL-cholesterol. No associations were found between long chain n-3 fatty acids or AA and any of the studied outcomes.

**Conclusions:**

Plasma phospholipid proportions of LA and ALA, biomarkers of vegetable oil intake, during childhood and adolescence were inversely associated with measures of obesity and cardiometabolic health in young adulthood, with a potential sex difference. These findings accord with short-term feeding trials suggesting a possible preventive role of LA on body fat accumulation.

## Introduction

Cardiovascular disease (CVD) is the leading cause of death globally, being responsible for one-third of all deaths [1]. Among important risk factors for CVD are obesity [2,3], high blood pressure (BP) [4,5], and dyslipidemia [6], which are greatly influenced by lifestyle choices and particularly diet.

A dietary component that has been hypothesized to be especially important in the development of obesity and other cardiometabolic risk factors is the quality and quantity of dietary fat. Specifically, dietary intake or supplementation with n-3 PUFAs have been suggested to regulate weight gain, positively influence BP, and contribute to a healthier blood lipid profile [7,8]. Although higher consumption of n-6 PUFA, especially resulting in a high n-6 to n-3 ratio, was earlier thought to promote inflammation and increase risk of CVD, recent studies have instead indicated that consumption of n-6 PUFAs is protective toward CVD [[9], [10], [11]].

Previous literature on diet early in life has reported conflicting results, and longitudinal studies are few and usually span only the first few years of life. A meta-analysis found that intake of fish or fish oil, rich in very long chain (VLC) n-3 PUFAs, in adults, mostly from the general population but also with diabetes or obesity, was associated with a very modest reduction in body weight, BMI, and waist circumference [12], whereas another meta-analysis including studies on children and adolescents with obesity found that n-3 fatty acid supplementation did not influence these outcomes [13]. In recent years, a meta-analysis compiled evidence indicating that the two essential fatty acids, α-linolenic acid (ALA, n-3) and linoleic acid (LA, n-6), which are mostly found in plant-based fats such as vegetable oils and nuts, may be more influential in regulating weight and cardiometabolic risk factors than marine fat [14].

Moreover, as obesity during childhood and early adulthood is largely predictive of obesity later in life, knowledge about how early consumption of dietary fat affects obesity and cardiometabolic risk factors later in life is highly desirable. This study aimed to investigate the relationship between plasma proportions of n-3 and n-6 PUFAs in childhood and adolescence and cardiometabolic risk factors such as obesity, body composition, BP, and blood lipids in young adulthood.

## Methods

### Study population

This study was conducted within the prospective birth cohort Barn Allergi Miljö Stockholm Epidemiologi (BAMSE), which has been previously described in detail [15,16]. The BAMSE birth cohort includes 4089 participants who were born between 1994 and 1996 in Stockholm County, Sweden. The participants were followed up repeatedly during infancy, childhood, and adolescence until 24 y of age through questionnaires enquiring about lifestyle factors, environmental exposures, and health outcomes, as well as with clinical examinations. At the 24-y follow-up (in 2016–2019), 3064 (75%) of the baseline participants answered the questionnaire, and 2271 (56%) attended the clinical examination. The Swedish Ethical Review Authority (approval number 2016/1380-31/2) approved the study. All participants provided written informed consent.

In this study, we included 688 participants who had answered the questionnaire and attended the clinical examination at 24 y of age and had data on fatty acids in plasma phospholipids at 8 or 16 y of age, excluding any participants who were pregnant when attending the 24-y examination (*n* = 1). The limiting factor was data availability on fatty acids in plasma phospholipids at 8 and 16 y of age, as this analysis was performed on a subsample of the cohort that had provided blood samples on both occasions and as some individuals had too small amounts of blood left to be analyzed [17]. Supplemental Figure 1 shows the participants included in this study.

### Exposure assessment

Fatty acids in plasma phospholipids collected at 8 and 16 y were measured through gas chromatography as previously described [18]. Briefly, the lipids were extracted with chloroform/methanol (2:1) with added butylated hydroxytoluene as an antioxidant. The phospholipids were isolated with thin-layer chromatography (TLC) plates (silica gel) eluted with the system petroleum ether/diethyl ether/acetic acid (81:18:1 by volume) and visualized in UV light. The lipid esters were methylated at 60°C overnight after the addition of H_2_SO_4_ (5%) in methanol. The fatty acid methyl esters were separated by gas-liquid chromatography on a Thermo TR-FAME 30-m column using an Agilent GLC 6890 N, autosampler 7683 and Agilent ChemStation (Agilent Technologies). The temperature was programmed to 150°C–260°C. The fatty acids were identified by comparing each peak’s retention time with fatty acid methyl ester standards Nu Check Prep (Elysian), and the 15 fatty acids were calculated as the area under the curve for the peaks on the chromatogram. Thus, they were expressed as proportions of total fatty acids comprising 15 different measured fatty acids in plasma (see Supplemental List 1). In the present study, we analyzed the n-3 fatty acid α-linolenic acid (ALA, 18:3n-3), the three VLC n-3 fatty acids EPA, docosapentaenoic acid (22:5n-3), and DHA, which were expressed as the sum of VLC n-3 fatty acids (∑VLC n-3 PUFAs), and the n-6 fatty acids linoleic acid (LA, 18:2n-6) and arachidonic acid (AA, 20:4n-6).

### Outcome assessment

All outcomes of the present study were assessed at ∼24 y of age. At the clinical examination, a research nurse measured the participants’ weight (kg) and height (cm), and BMI was calculated as weight/height (kg/m^2^). Overweight was defined as a BMI ≥ 25 and <30 kg/m^2^ and obesity as BMI ≥ 30 kg/m^2^. Waist circumference was measured with a tape measurer to the nearest centimeter. A Tanita MC 780 body composition monitor was used to estimate fat mass percentage (%) with bioelectric impedance analysis. Systolic and diastolic BP was measured in triplicate after 5 min of rest in a sitting position, and the average of the 3 measurements obtained with Omron HBP-1300 was used. A venous blood sample was collected from the participants to obtain information about blood lipids. Analyses of blood lipids were performed by the Clinical Chemistry department of Karolinska University Hospital, Stockholm, using a Cobas 8000 c701 (Roche Diagnostics). The measured blood lipids were triglycerides, total cholesterol, HDL-cholesterol), and LDL-cholesterol). No samples were outside the ranges of detection for the measurement of triglycerides (0.1–10.0 mmol/L), total cholesterol (0.1–20.7 mmol/L), or HDL-cholesterol (0.08–3.12 mmol/L for some of the measurements and 0.08–3.88 mmol for the remaining measurements). The coefficients of variation for the measurement of triglycerides, total cholesterol, and HDL-cholesterol were 6%, 4%, and 7%, respectively, and the laboratory is accredited for these measurements and performs daily quality control routines with high and low concentrations. LDL-cholesterol was calculated according to Friedewald’s equation through the accredited measurements of total cholesterol, HDL-cholesterol, and triglycerides. The calculation was not performed if triglycerides >4.0 mmol/L (2 samples) and only 1 sample had the calculated LDL-cholesterol concentration < 0.2 mmol/L and was noted as 0 mmol/L.

### Covariates

Information on covariates used in the present study was obtained mainly from either the baseline questionnaire or from the questionnaire at the 24-y follow-up; additionally, fiber intake was assessed at 8 and 16 y of age. The variables obtained from the baseline questionnaire were parental socioeconomic status (SES) at baseline, categorized as the parents being either professional or nonprofessional workers according to Statistics Sweden [“Socioeconomic division (SEI); Reports on Statistical Coordination 1982:4”], parental smoking at baseline, categorized as any parent smoking ≥1 cigarette/day or not, maternal smoking during pregnancy (mother smoking ≥1 cigarette per day at any time point in pregnancy or not), and birth weight (in grams, as a continuous variable). The variables from the questionnaire at 24 y of age were occupation (studying, employed, or other), education (studies after secondary school or not), current smoking (yes or no), current snuff use (yes or no), and sedentary level (sitting time per day excluding sleep: >10 h, between 7 and 9 h, ≤6 h), all at 24 y. Energy-adjusted dietary fiber intake (in grams, as a continuous variable) and total energy intake were estimated through a food frequency questionnaire at 8 and 16 y of age [19]. Phospholipid plasma proportions of palmitic acid (16:0), stearic acid (18:0), and oleic acid (18:1n-9) at 8 and 16 y were assessed as previously described [18].

### Statistical analyses

All analyses were performed using Stata SE version 17 (StataCorp), and a *P* value below 0.05 was considered statistically significant. Participant characteristics were summarized with descriptive statistics, and the difference between female and male participants in exposure, outcome, and covariate variables was assessed via Student’s *t* test for continuous variables and Pearson’s chi-square test for categorical variables.

Initially, bivariate associations between fatty acids and different outcomes were assessed with Spearman’s correlation. Associations between fatty acids and outcomes were also visually inspected through scatterplots fitted with Lowess lines, which did not show indications of nonlinear relationships. Therefore, the associations were modeled using multivariable-adjusted linear regression, with PUFA proportions as continuous variables analyzed separately for exposure at 8 and 16 y. The crude models were unadjusted. The adjusted models included sex, parental SES, and parental smoking at baseline, maternal smoking during pregnancy, birth weight, dietary fiber intake at 8 y (in models of plasma PUFAs at 8 y) or 16 y (in models of plasma PUFAs at 16 y), and occupation, education, smoking, snuff use, and sedentary level at 24 y, which were chosen a priori. In the adjusted models we included participants with complete covariate data. A multiplicative interaction term between plasma PUFAs and sex was computed. As the *P* value of this interaction term was <0.05 in several models, and due to the statistically significant difference in outcome variables between male and female participants, all subsequent models were stratified by sex. As a sensitivity analysis, we additionally adjusted for total energy intake at 8 y (in models of plasma PUFAs at 8 y) or 16 y (in models of plasma PUFAs at 16 y) as a way to adjust for overall dietary intake. We also conducted another sensitivity analysis adjusting for the phospholipid plasma proportions of 3 lipogenic fatty acids, palmitic, stearic, and oleic acids, at 8 and 16 y.

To follow-up on the associations that were found, a logistic regression analysis was conducted to calculate the odds ratio (OR) of being overweight or obese at 24 y (BMI ≥ 25) between female participants with plasma ALA and LA proportions at 8 and 16 y above the median compared with those with PUFA proportions below the median. The logistic regression models were adjusted for the same covariates as the linear regression models. The results were reported as effect coefficients for linear regression and odds ratio for logistic regression, with their corresponding 95% confidence interval (CI).

## Results

### Characteristics

The characteristics of the female and male participants of the present study are summarized in Table 1. Most of the participants were born to nonsmoking parents who were professional workers. At the 24-y follow-up, the participants were quite evenly split between studying and working, and ∼20% were daily or occasional smokers, 76% had a normal weight or underweight, whereas 19% had a BMI classified as overweight and 5% had obesity. There were some differences between males and females. Female participants were more often studying and had attained university education than male participants. Females smoked more often, whereas males more often used snuff. Females had a higher intake of dietary fiber at both 8 and 16 y of age. Males had higher birth weight than females, whereas the other baseline characteristics, such as parental SES, paternal smoking at baseline, and maternal smoking during pregnancy did not differ by sex. Regarding the cardiometabolic health factors measured at 24 y, apart from BMI and LDL-cholesterol, the means of all other outcomes differed between males and females. Instead, the mean proportions of the different plasma PUFAs at 8 and 16 y were comparable among males and females with the exception for AA, which had higher mean proportions in males at both time points (Table 2). The full fatty lipid profiles are shown in Supplemental Table 1.

**TABLE 1:** Characteristics of the 688 study participants by sex, 408 females and 280 males.

Characteristics	Females			Males		
	*n*	Mean (or %)	SD	*n*	Mean (or %)	SD
At baseline						
Parental SES	405			278		
Professional worker	359	89%		246	88%	
Nonprofessional worker	47	11%		32	12%	
Birth weight (g)	408	3468	581	280	3558	523
Parental smoking at baseline	405			279		
No	330	81%		226	81%	
Yes	75	19%		53	19%	
Maternal smoking during pregnancy	407			280		
No	359	88%		255	91%	
Yes	48	12%		25	9%	
Diet						
Dietary fiber intake at 8 y (g)	408	18.6	3.69	280	17.8	3.86
Dietary fiber intake at 16 y (g)	399	19.1	5.61	273	17.0	4.27
Total energy intake at 8 y (kcal)	408	1882	433	280	1979	460
Total energy intake at 16 y (kcal)	403	1714	605	276	2181	890
At the 24 y follow-up						
Age (y)	408	22.6	0.53	280	22.6	0.56
Occupation	408			280		
Studying	242	59%		139	50%	
Employed	149	37%		113	40%	
Other	17	4%		28	10%	
Education	408			280		
University	187	46%		84	30%	
Secondary school	221	54%		196	70%	
Smoking	408			280		
No	315	77%		236	84%	
Yes	93	23%		44	16%	
Snuff	408			280		
No	384	94%		216	77%	
Yes	24	6%		64	23%	
Sedentary level (h)	407			278		
≤6	164	40%		102	37%	
7–9	145	36%		85	31%	
≥10	97	24%		91	33%	
Outcomes at 24-y follow-up						
BMI (kg/m^2^)^1^	408	22.9	3.87	280	23.5	3.53
BMI categories (kg/m^2^)^1^	408			280		
<25	317	78%		207	74%	
≥25 and <30	75	18%		57	20%	
≥30	16	4%		16	6%	
Waist circumference (cm)^1^	408	75	8.65	278	84	9.19
Fat mass % (%)^1^	400	27	6.09	275	16	6.19
Systolic BP (mmHg)^1^	408	117	8.7	280	130	11
Diastolic BP (mmHg)^1^	408	75	7.6	280	77	8.6
Triglycerides (mmol/L)^1^	402	0.98	0.43	280	1.18	0.66
Total cholesterol (mmol/L)^1^	402	4.28	0.81	280	4.09	0.77
HDL-cholesterol (mmol/L)^1^	402	1.72	0.41	280	1.4	0.32
LDL-cholesterol (mmol/L)^1^	401	2.12	0.70	279	2.16	0.72

1 At the clinical visit of the 24-y follow-up.

**TABLE 2:** Mean plasma phospholipid proportions of PUFA at 8 and 16 y of age in female and male participants.

Plasma PUFA proportions^1^	Females			Males			*P* value^2^
	*n*	%	SD	*n*	%	SD	
ALA 18:3n-3 at 8 y (%)	408	0.24	0.065	278	0.25	0.073	0.087
ALA 18:3n-3 at 16 y (%)	406	0.30	0.087	280	0.29	0.078	0.054
∑VLC n-3 PUFA at 8 y (%)	408	3.36	0.99	279	3.47	1.06	0.15
∑VLC n-3 PUFA at 16 y (%)	406	5.59	1.43	280	5.53	1.18	0.57
LA 18:2n-6 at 8 y (%)	408	21.36	1.82	279	21.21	2.04	0.34
LA 18:2n-6 at 16 y (%)	406	21.99	2.04	280	21.93	2.11	0.74
AA 20:4n-6 at 8 y (%)	408	5.55	1.35	279	5.78	1.29	0.023
AA 20:4n-6 at 16 y (%)	406	8.74	1.30	280	9.23	1.24	<0.001

AA, arachidonic acid; ALA, α-linolenic acid; LA, linoleic acid; SD, standard deviation; ∑VLC n-3 PUFA, sum of very long chain n-3 PUFAs.

1 Proportion of 15 total fatty acids measured in plasma.

2 Comparing female and male participants using *t* test.

The proportions of the specific plasma phospholipid PUFAs at 8 y were weakly to moderately associated with their proportions at 16 y (rho from 0.17 to 0.35). The correlations between the specific PUFA proportions at both 8 and 16 y varied from no association, to moderate, to high in the case of ∑VLC n-3 PUFAs and AA at 8 y (rho = 0.81, *P* value < 0.001) (Supplemental Table 2).

The participants included in the present study (*n* = 688) were representative of the whole BAMSE cohort (*n* = 4089) in many aspects, displaying comparable baseline characteristics (Supplemental Table 3). However, there was a somewhat higher representation of female participants (59% compared with 49%) and participants originating from higher socioeconomic status (89% compared with 83%) compared with the whole BAMSE cohort, reflecting the sex distribution in participation in the 24-y follow-up to a larger extent. The cardiometabolic outcomes measured in the follow-up at 24 y were similar to that of all BAMSE individuals taking part in the clinical examination at the 24-y follow-up (*n* = 2270) (Supplemental Table 4).

### Plasma PUFA proportions in relation to obesity markers

The results of the sex-stratified adjusted linear regression models with BMI, waist circumference, and fat mass percentage are presented in Table 3. The crude and adjusted models obtained almost identical effect coefficients (Supplemental Table 5), and therefore only the adjusted models’ results are reported in the following tables (TABLE 3, TABLE 4, TABLE 5).

**TABLE 3:** Multivariable-adjusted linear regression models of plasma PUFA proportions at 8 and 16 y and BMI, waist circumference, and fat mass % at 24 y.

Plasma PUFA proportions^1^	Females		Males	
	*n*	B (95% CI)	*n*	B (95% CI)
BMI				
ALA				
8 y	399	−1.6 (−7.5, 4.3)	273	−0.11 (−5.8, 5.7)
16 y	388	−6.1 (−11, −1.5)	269	4.4 (−1.2, 9.9)
∑VLC n-3				
8 y	399	0.17 (−0.23, 0.57)	274	−0.22 (−0.63, 0.19)
16 y	388	0.030 (−0.25, 0.31)	269	0.029 (−0.34, 0.40)
LA				
8 y	399	−0.39 (−0.60, −0.19)	274	−0.038 (−0.25, 0.17)
16 y	388	−0.35 (−0.54, −0.17)	269	−0.098 (−0.30, 0.11)
AA				
8 y	399	0.14 (−0.15, 0.43)	274	−0.028 (−0.36, 0.31)
16 y	388	0.17 (−0.13, 0.47)	269	0.19 (−0.16, 0.54)
Waist circumference				
ALA				
8 y	399	−8.6 (−22, 4.6)	271	−1.6 (−17, 13)
16 y	388	−14 (−24, −4.3)	267	11 (−3.1, 26)
∑VLC n-3				
8 y	399	0.039 (−0.85, 0.93)	272	−0.65 (−1.7, 0.42)
16 y	388	0.19 (−0.44, 0.82)	267	−0.17 (−1.1, 0.79)
LA				
8 y	399	−0.83 (−1.30, −0.36)	272	0.054 (−0.49, 0.60)
16 y	388	−0.95 (−1.37, −0.53)	267	−0.31 (−0.84, 0.22)
AA				
8 y	399	−0.053 (−0.70, 0.59)	272	−0.34 (−1.2, 0.54)
16 y	388	0.24 (−0.43, 0.92)	267	0.16 (−0.75, 1.1)
Fat mass %				
ALA				
8 y	391	−4.0 (−13, 5.3)	268	−0.95 (−11, 9.3)
16 y	380	−10 (−17, −2.8)	264	3.4 (−6.4, 13)
∑VLC n-3				
8 y	391	0.23 (−0.40, 0.86)	269	−0.52 (−1.3, 0.22)
16 y	380	0.11 (−0.34, 0.56)	264	−0.23 (−0.88, 0.43)
LA				
8 y	391	−0.63 (−0.96, −0.30)	269	−0.18 (−0.55, 0.19)
16 y	380	−0.55 (−0.85, −0.25)	264	−0.22 (−0.58, 0.14)
AA				
8 y	391	0.079 (−0.38, 0.54)	269	−0.081 (−0.69, 0.53)
16 y	380	0.13 (−0.34, 0.61)	264	0.32 (−0.29, 0.94)

AA, arachidonic acid; ALA, α-linolenic acid; CI, confidence interval; LA, linoleic acid; ∑VLC n-3 PUFA, sum of very long chain n-3 PUFAs.

1 Models adjusted for parental socioeconomic status at baseline (professional or nonprofessional worker), occupation at 24 y (studying, employed, other), education at 24 y (studies after secondary school or no), smoking at 24 y (yes or no), snus at 24 y (yes or no), sedentary level at 24 y (≤6, 7–9, or ≥10 h), birth weight (g), parental smoking at baseline (yes or no), maternal smoking during pregnancy (yes or no), dietary fiber intake at 8 y (g, in models of plasma PUFA at 8 y) or dietary fiber intake at 16 y (g, in models of plasma PUFA at 16 y).

In female participants, we observed an inverse association between LA at both time points and BMI, waist circumference, and fat mass percentage. An increase of 1% of the plasma proportion of LA at either 8 or 16 y corresponded to a lower BMI at 24 y of 0.39 and 0.35, respectively, a smaller waist circumference of 0.83 and 0.95 cm, and a lower fat mass percentage of 0.63% and 0.55% (Table 3). Indeed, females with plasma proportions of LA above the median both at 8 (21.36%) and 16 y (21.86%) had nearly 50% lower odds of being overweight or obese at 24 y compared with those with LA proportions below the median [OR: 0.58 (0.35, 0.97) and OR: 0.54 (0.32, 0.90), respectively; Supplemental Figure 2)]. Moreover, increasing plasma proportions of ALA at 16 y were associated with lower measures of all three obesity-related outcomes at 24 y (Table 3). Correspondingly, plasma proportions of ALA at 16 y above the median of 0.28% were also associated with lower odds of overweight/obesity at 24 y than proportions below the median [OR: 0.56 (0.34, 0.93); Supplemental Figure 2)]. No associations were observed between ∑VLC n-3 PUFA or AA at either time point or any measure of obesity.

No associations were observed between any of the plasma fatty acids and obesity markers in male participants. Indeed, there was an indication of an interaction between plasma ALA and LA proportions and sex. There was a statistically significant interaction between ALA at 16 y and sex in models with all 3 obesity markers as the outcome (*P*_int_ = between 0.001 and 0.016) and between LA at 8 y and sex in models with BMI and waist circumference as the outcome (*P*_int_ = 0.019 and 0.014, respectively).

Adjusting for total energy intake at 8 or 16 y of age did not affect any of the observed associations (Supplemental Table 6). When adjusting for plasma proportions of palmitic, stearic, and oleic acids at 8 and 16 y, the inverse associations between ALA and LA and obesity markers in females did not change markedly. However, we also observed a positive association between ∑VLC n-3 PUFAs and AA and BMI in females, as well as a positive association between ALA and BMI and waist circumference in males (Supplemental Table 7).

### Plasma PUFA proportions in relation to BP

In females, LA at both 8 and 16 y was inversely associated with diastolic BP [B: −0.46 (−0.87, −0.044) and B: −0.40 (−0.78, −0.022), respectively], and moreover LA at 8 y was inversely associated with systolic BP at 24 y [B: −0.50 (−0.98, −0.027)] (Table 4). No statistically significant associations were observed between other plasma PUFA proportions at either time point and BP.

**TABLE 4:** Multivariable-adjusted linear regression models of plasma PUFA proportions at 8 and 16 y and systolic and diastolic blood pressure at 24 y.^1^

Plasma PUFA proportions	Females		Males	
	*n*	B (95% CI)	*n*	B (95% CI)
Systolic blood pressure				
ALA				
8 y	399	−7.4 (−21, 5.9)	273	2.9 (−16, 22)
16 y	388	3.1 (−7.3, 13)	269	1.4 (−17, 20)
∑VLC n-3				
8 y	399	−0.15 (−1.1, 0.75)	274	0.10 (−1.2, 1.4)
16 y	388	0.43 (−0.20, 1.1)	269	−0.43 (−1.6, 0.78)
LA				
8 y	399	−0.50 (−0.98, −0.027)	274	0.087 (−0.59, 0.76)
16 y	388	−0.40 (−0.84, 0.030)	269	0.34 (−0.33, 1.0)
AA				
8 y	399	−0.21 (−0.86, 0.45)	274	0.033 (−1.1, 1.1)
16 y	388	−0.17 (−0.85, 0.52)	269	−0.11 (−1.3, 1.0)
Diastolic blood pressure				
ALA				
8 y	399	−5.0 (−17, 6.6)	273	−2.7 (−17, 12)
16 y	388	−1.9 (−11, 7.2)	269	−12 (−26, 2.0)
∑VLC n-3				
8 y	399	−0.53 (−1.3, 0.25)	274	0.26 (−0.78, 1.3)
16 y	388	0.17 (−0.39, 0.73)	269	0.16 (−0.78, 1.1)
LA				
8 y	399	−0.46 (−0.87, −0.044)	274	−0.23 (−0.75, 0.29)
16 y	388	−0.40 (−0.78, −0.022)	269	0.25 (−0.77, 0.27)
AA				
8 y	399	−0.32 (−0.89, 0.24)	274	0.12 (−0.72, 0.97)
16 y	388	0.13 (−0.47, 0.73)	269	0.53 (−0.36, 1.4)

AA, arachidonic acid; ALA, α-linolenic acid; CI, confidence interval; LA, linoleic acid; ∑VLC n-3 PUFA, sum of very long chain n-3 PUFAs.

1 Models adjusted for parental socioeconomic status at baseline (professional or nonprofessional worker), occupation at 24 y (studying, employed, other), education at 24 y (studies after secondary school or no), smoking at 24 y (yes or no), snus at 24 y (yes or no), sedentary level at 24 y (≤6, 7–9, or ≥10 h), birth weight (g), parental smoking at baseline (yes or no), maternal smoking during pregnancy (yes or no), dietary fiber intake at 8 y (g, in models of plasma PUFA at 8 y) or dietary fiber intake at 16 y (g, in models of plasma PUFA at 16 y).

No associations were found between any of the plasma fatty acids and BP among male participants. However, there was no clear indication of effect modification by sex in models of plasma PUFAs and BP; the only statistically significant interaction with sex was with LA at 16 y in models of systolic BP (*P*_int_ = 0.047).

Sensitivity analyses showed no differences in any of the observed associations after adjustment for total energy intake at 8 or 16 y (Supplemental Table 8). In contrast, no associations could be observed between any PUFAs and BP after adjustment for palmitic, stearic, and oleic acid proportions (Supplemental Table 9).

### Plasma PUFA proportions in relation to blood lipids

In females, we observed that LA at 16 y of age was inversely associated with the concentration of triglycerides [B: −0.025 (−0.047, −0.0034)], total cholesterol [B: −0.040 (−0.081, −0.000056)], and LDL-cholesterol [B: −0.044 (−0.079, −0.0099)] at 24 y of age (Table 5). Moreover, ALA at 16 y was inversely associated with LDL-cholesterol (B: −0.88 [−1.7, −0.056]). Neither ∑VLC n-3 PUFAs nor AA at either time point was associated with blood lipids at 24 y. No statistically significant associations were observed among the male participants. However, there was an indication of an inverse association between ALA at 16 y and LDL-cholesterol in males [B: −1.0 (−2.1, 0.11)] similar to what we observed in females (Table 5). Instead, we found evidence of statistically significant interactions between ALA at 8 y and ALA at 16 y and sex in models of triglycerides (*P*_int_ = 0.041) and HDL-cholesterol (*P*_int_ = 0.032), respectively.

**TABLE 5:** Multivariable-adjusted linear regression models of plasma PUFA proportions at 8 and 16 y and blood lipids at 24 y.^1^

Plasma PUFA proportions	Females		Males	
	*n*	B (95% CI)	*n*	B (95% CI)
Triglycerides				
ALA				
8 y	393	0.54 (−0.13, 1.2)	273	−0.81 (−1.9, 0.28)
16 y	382	−0.25 (−0.78, 0.27)	269	0.62 (−0.42, 1.7)
∑VLC n-3				
8 y	393	−0.0037 (−0.049, 0.042)	274	−0.0051 (−0.083, 0.073)
16 y	382	0.023 (−0.0093, 0.055)	269	−0.036 (−0.11, 0.034)
LA				
8 y	393	0.0017 (−0.023, 0.026)	274	−0.0037 (−0.043, 0.036)
16 y	382	−0.025 (−0.047, −0.0034)	269	0.014 (−0.025, 0.053)
AA				
8 y	393	−0.0057 (−0.039, 0.027)	274	−0.016 (−0.080, 0.047)
16 y	382	−0.0031 (−0.038, 0.032)	269	−0.028 (−0.094, 0.038)
Total cholesterol				
ALA				
8 y	393	0.68 (−0.57, 1.9)	273	0.16 (−1.1, 1.4)
16 y	382	−0.54 (−1.5, 0.43)	269	−1.0 (−2.2, 0.22)
∑VLC n-3				
8 y	393	−0.034 (−0.12, 0.050)	274	0.0081 (−0.081, 0.097)
16 y	382	0.012 (−0.048, 0.072)	269	−0.019 (−0.10, 0.063)
LA				
8 y	393	−0.020 (−0.065, 0.025)	274	−0.030 (−0.075, 0.015)
16 y	382	−0.040 (−0.081, −0.000056)	269	−0.0030 (−0.048, 0.042)
AA				
8 y	393	−0.049 (−0.11, 0.012)	274	0.011 (−0.062, 0.083)
16 y	382	−0.052 (−0.12, 0.011)	269	−0.0064 (−0.083, 0.071)
HDL-cholesterol				
ALA				
8 y	393	0.53 (−0.098, 1.1)	273	0.012 (−0.52, 0.54)
16 y	382	0.41 (−0.082, 0.89)	269	−0.30 (−0.81, 0.21)
∑VLC n-3				
8 y	393	−0.017 (−0.059, 0.025)	274	0.0099 (−0.028, 0.047)
16 y	382	0.0063 (−0.024, 0.036)	269	0.011 (−0.023, 0.045)
LA				
8 y	393	0.019 (−0.0034, 0.042)	274	0.00034 (−0.019, 0.019)
16 y	382	0.018 (−0.0022, 0.039)	269	0.0031 (−0.016, 0.022)
AA				
8 y	393	−0.016 (−0.047, 0.014)	274	−0.0028 (−0.033, 0.028)
16 y	382	−0.0037 (−0.036, 0.029)	269	−0.0012 (−0.034, 0.031)
LDL-cholesterol				
ALA				
8 y	392	−0.11 (−1.2, 0.96)	272	0.29 (−0.87, 1.5)
16 y	381	−0.88 (−1.7, −0.056)	268	−1.0 (−2.1, 0.11)
∑VLC n-3				
8 y	392	−0.024 (−0.096, 0.047)	273	0.0013 (−0.082, 0.084)
16 y	381	−0.011 (−0.062, 0.040)	268	−0.032 (−0.11, 0.043)
LA				
8 y	392	−0.031 (−0.070, 0.0077)	273	−0.028 (−0.070, 0.014)
16 y	381	−0.044 (−0.079, −0.0099)	268	−0.0028 (−0.045, 0.039)
AA				
8 y	392	−0.029 (−0.081, 0.023)	273	0.015 (−0.052, 0.083)
16 y	381	−0.049 (−0.10, 0.0055)	268	−0.0022 (−0.073, 0.069)

AA, arachidonic acid; ALA, α-linolenic acid; CI, confidence interval; ∑VLC n-3 PUFA, sum of very long chain n-3 PUFAs.

1 Models adjusted for parental socioeconomic status at baseline (professional or nonprofessional worker), occupation at 24 y (studying, employed, other), education at 24 y (studies after secondary school or no), smoking at 24 y (yes or no), snus at 24 y (yes or no), sedentary level at 24 y (≤6, 7–9, or ≥10 h), birth weight (g), parental smoking at baseline (yes or no), maternal smoking during pregnancy (yes or no), dietary fiber intake at 8 y (g, in models of plasma PUFA at 8 y), or dietary fiber intake at 16 y (g, in models of plasma PUFA at 16 y).

In sensitivity analysis, adjustment for total energy intake at 8 or 16 y did not affect any of the observed associations (Supplemental Table 10). However, after adjusting for the proportions of palmitic, stearic, and oleic acids, the associations in females for LA at 16 y in relation to triglycerides, total cholesterol, and LDL-cholesterol, as well as for ALA at 16 y in relation to LDL-cholesterol became nonsignificant. Instead, we observed a positive association between ALA at 8 y and total cholesterol in females, a positive association between ALA at 16 y and LA at 8 y and HDL-cholesterol in females, and a positive association between AA at 8 y and LDL-cholesterol in males (Supplemental Table 11).

## Discussion

This is the first longitudinal study investigating the relationship between repeated measurements of biomarkers of PUFA intake in childhood and adolescence and markers of cardiometabolic health in young adulthood. In females, plasma phospholipid proportions of LA and ALA at 8 and 16 y of age were inversely associated with obesity markers at 24 y. Moreover, also in females, higher plasma proportions of LA were associated with lower BP as well as a better blood lipid profile in adulthood, with lower triglycerides, total cholesterol, and LDL-cholesterol, and ALA at 16 y was associated with lower LDL-cholesterol at 24 y of age. No significant associations were observed in males. Interestingly, we found no association between plasma proportions of VLC n-3 PUFAs and any of the studied cardiometabolic risk factors.

The observed inverse association between the essential fatty acids LA and ALA, but not with the long chain marine n-3 PUFAs, with obesity are consistent with a recent review of observational and intervention studies, mainly in adults [14]. LA has previously been inversely associated with obesity measures in short-term randomized controlled trials (RCTs) as well as observational studies [[20], [21], [22]]. For example, in an overfeeding study of normal-weight adults, LA-rich sunflower oil for 7 wk led to less accumulation of total fat, liver fat, and visceral fat, versus an isocaloric overfeeding with palm oil, rich in saturated fat [20]. In a large cross-sectional study of Swedish 60-y-old male and female participants, serum LA and ALA, as well as DHA, were linked to lower abdominal obesity [22]. However, knowledge is scarce in children, prospective data in particular. Two Danish longitudinal studies found no association between dietary intake of marine fat and long chain n-3 PUFAs during childhood, respectively, and measures of obesity; however, associations with n-6 PUFAs were not assessed [23,24]. A cross-sectional study of American school-aged children found an association between dietary PUFAs (both n-3, composed primarily of ALA, and n-6 PUFAs) and higher lean mass and lower body fat % [25]. Different from our results, a Chilean prospective study found an inverse association between DHA and EPA in childhood and BMI z-score in adolescence, but no association with LA or ALA [26]. The scarcity of longitudinal studies with a long follow-up time warrants the need for more.

In contrast to our results of no association, an association between high doses of the long chain n-3 PUFAs DHA and EPA and lower BP has repeatedly been reported in supplementation studies both in adults [27,28] and in children [29]. Conversely, there is less and conflicting evidence for an association between LA and lower BP [30,31], including a Cochrane report not supporting this association in adults [32]. A Finnish population-based observational study with a follow-up time of 27 y found that higher proportions of n-6 PUFAs measured in plasma cholesterol esters during childhood and adolescence were associated with lower BP [33]. In our study, we did not observe any association between ∑VLC n-3 PUFAs and BP. Instead, we reported a tenuous association in female participants between plasma proportions of LA and lower systolic and diastolic BP at 24 y. Many of the previous studies reporting associations between PUFAs and BP were supplementation studies, or had middle-aged participants. It is possible that the weakness of the associations found in the present study, and the lack of an association between ∑VLC n-3 PUFAs and BP, may be because the intake of the n-3 fatty acids was not as high as the one achievable in supplementation studies. Moreover, the participants of this study were in young adulthood. At 24 y of age, only 7% of the participants had a systolic BP that is considered elevated (>140 mmHg), almost all of them male, and <4% had a diastolic BP >90 mmHg. In comparison, 24% were classified as overweight or obese.

Our reported inverse association between plasma LA and triglycerides, total cholesterol, and LDL-cholesterol, but not HDL-cholesterol, is consistent with the results of a Swedish RCT, in which individuals with hyperlipidemia replaced some of their intake of dairy fat with rapeseed oil, rich in LA and ALA [34]. Earlier epidemiological and RCTs have also reported a lack of association between increased consumption of rapeseed oil and HDL-cholesterol, unlike the well-documented association with lower LDL-cholesterol [[35], [36], [37]]. It has been suggested that a higher intake of PUFAs leads to lower serum LDL-cholesterol concentrations through the modulation of the levels of the enzyme proprotein convertase subtilisin/kexin type 9, which normally breaks down hepatic LDL-cholesterol receptors and whose inhibition results in higher hepatic uptake of LDL-cholesterol, lowering serum levels [21,38].

Although the exact mechanism of how consumption of PUFAs may result in reduced obesity is not known, it may be due to diet-induced thermogenesis and fat oxygenation following their ingestion [39,40]. Indeed, recent findings have suggested a higher whole-body oxidation of dietary LA as compared with saturated palmitic acid [41]. Furthermore, higher plasma proportions of LA have been directly associated with higher resting energy expenditure [42]. PUFAs may also inhibit de novo lipogenesis, as LA has been found to reduce lipogenic markers, which in turn were closely associated with an LA-induced reduction of liver fat content [20,21]. The degree of adiposity may be a mediator in the associations between LA and BP and blood lipids. Moreover, in this cohort, it was found that overweight and obesity were associated with an inflammatory protein profile, which has been related to an increased risk of development of cardiometabolic disease [43].

We found that the associations between LA and ALA and markers of cardiometabolic health were statistically significant only in females, further supported by the positive interaction observed between sex and plasma proportions of LA and ALA for some of the outcomes. Nevertheless, a possible explanation could be that several of the studied outcomes have different distribution in males and females. Moreover, our study included more females than males, giving higher statistical power among female participants. No consistent differences between the sexes have been reported in the previous literature, but this may be explained by different susceptibility among males and females across the life course.

Important strengths of this study are the population-based longitudinal design, the long follow-up period, and repeated individual measurements of exposure biomarkers for a large number of participants. Another strength is that three different obesity markers were available, which showed the consistency of our results, as well as data on total energy intake and on plasma proportions of 15 different fatty acids. Plasma phospholipid proportions of PUFAs, especially those of LA, DHA, and EPA, are reliable biomarkers of their dietary intake [44], and their use reduces exposure misclassification in comparison with food frequency questionnaires. However, plasma proportions of PUFAs do not only reflect the intake through the diet but also depend on endogenous metabolism. Moreover, plasma proportions of PUFAs can also quickly reflect short-term changes in diet in a matter of days [45], and therefore could misclassify the long-term diet of some participants. Only a subset of the BAMSE cohort had data on plasma PUFAs and could be included in this study; however, this subset was representative of the whole cohort with regard to most background characteristics, with the exception of an over-representation of females, and representative of all the participants of the 24 y follow-up regarding the health outcomes studied herein. These results were obtained in a population-based cohort with a prevalence of overweight and obesity comparable with that of the Swedish general population at the same age span [46], and they may be relevant to many young adults living in large parts of the industrialized world.

In conclusion, this longitudinal study supports short-term feeding trials and suggests that the choice of dietary fat already in childhood and adolescence may play a role in preventing body fat accumulation and cardiometabolic health risk in adulthood. Our findings further support the current guidelines in adults [47] recommending the intake of plant-based fats such as rapeseed and sunflower oil, rich in LA and ALA.

## Conflict of interest

The authors declare that they have no known competing financial interests or personal relationships that could have appeared to influence the work reported in this article.

## Data Availability

Data described in the manuscript, code book, and analytic code will be made available upon request pending an approved application and relevant agreements.
