# Supplementary material for: Biomarkers of dietary PUFA intake in childhood and adolescence in relation to cardiometabolic risk factors in young adulthood: a prospective cohort study in Sweden
Source: Am J Clin Nutr. 2025 Feb 7;121(3):558–66. doi: 10.1016/j.ajcnut.2024.11.029 (PMC11923371; doi:10.1016/j.ajcnut.2024.11.029)
Supplement: Multimedia component 1 [file mmc1.docx]

**Supplemental material**

**Biomarkers of dietary polyunsaturated fatty acid intake in childhood and adolescence in relation to cardiometabolic risk factors in young adulthood: a prospective cohort study.**

Annachiara Malin Igra *et al.*

**Table of contents**

**Supplemental List 1.** List of measured fatty acids.

**Supplemental Table 1.** Plasma phospholipid proportions of fatty acids.

**Supplemental Table 2.** Spearman’s rank correlations between PUFAs.

**Supplemental Table 3.** Comparison of baseline characteristics between participants of this study and the entire BAMSE cohort.

**Supplemental Table 4.** Comparison of cardiometabolic outcomes at 24 years between the participants of this study and all participants to the clinical examination at 24 years.

**Supplemental Table 5.** Crude and multivariable-adjusted models of PUFA proportions at 8 and 16 years and BMI at 24 years.

**Supplemental Table 6.** Sensitivity analysis of linear regression models with obesity markers as outcome additionally adjusted for total energy intake at 8 and 16 years.

**Supplemental Table 7.** Sensitivity analysis of linear regression models with obesity markers as outcome additionally adjusted for proportions of palmitic, stearic and oleic acids at 8 and 16 years.

**Supplemental Table 8.** Sensitivity analysis of linear regression models with blood pressure as outcome additionally adjusted for total energy intake at 8 and 16 years.

**Supplemental Table 9.** Sensitivity analysis of linear regression models with blood pressure as outcome additionally adjusted for proportions of palmitic, stearic and oleic acids at 8 and 16 years.

**Supplemental Table 10.** Sensitivity analysis of linear regression models with blood lipids as outcome additionally adjusted for total energy intake at 8 and 16 years.

**Supplemental Table 11.** Sensitivity analysis of linear regression models with blood lipids as outcome additionally adjusted for proportions of palmitic, stearic and oleic acids at 8 and 16 years.

**Supplemental Figure 1.** Flowchart of study participants.

**Supplemental Figure 2.** Odds ratio of being overweight or obese in females with LA and ALA above vs below the median.

| **Supplemental List 1.** List of measured fatty acids.  The 15 fatty acids measured in plasma at 8 and 16 years of age were:  1. Myristic acid (14:0)  2. Pentadecyclic acid (15:0)  3. Palmitic acid (16:0)  4. Palmitoleic acid (16:1)  5. Margaric acid (17:0)  6. Stearic acid (18:0)  7. Oleic acid (18:1)  8. Linoleic acid (LA, 18:2)  9. γ-Linolenic acid (18:3)  10. α-Linolenic acid (ALA, 18:3n-3)  11. Dihomo-γ-linoleic acid (20:3)  12. Arachidonic acid (AA, 20:4)  13. Eicosapentaeonic acid (EPA, 20:5n-3)  14. Docosapentaeonic acid (DPA, 22:5n-3)  15. Docosahexaeonic acid (DHA, 22:6n-3) |
| --- |

| **Supplemental Table 1.** Mean plasma phospholipid proportions of all 15 measured fatty acids at 8 and 16 years of age in female and male participants. | | | | | | |
| --- | --- | --- | --- | --- | --- | --- |
| Plasma proportions of fatty acids (%) | Females | | | Males | | |
|  | n | % | SD | n | % | SD |
| Myristic acid (14:0) at 8y | 408 | 0.49 | 0.11 | 279 | 0.48 | 0.12 |
| Myristic acid (14:0) at 16y | 406 | 0.41 | 0.092 | 280 | 0.37 | 0.081 |
| Pentadecyclic acid (15:0) at 8y | 408 | 0.27 | 0.041 | 279 | 0.27 | 0.041 |
| Pentadecyclic acid (15:0) at 16y | 406 | 0.33 | 0.085 | 280 | 0.34 | 0.092 |
| Palmitic acid (16:0) at 8y | 408 | 33.21 | 1.6 | 279 | 33.17 | 1.64 |
| Palmitic acid (16:0) at 16y | 406 | 30.83 | 1.66 | 280 | 30.11 | 1.27 |
| Palmitoleic acid (16:1) at 8y | 408 | 0.5 | 0.14 | 279 | 0.49 | 0.17 |
| Palmitoleic acid (16:1) at 16y | 406 | 0.44 | 0.12 | 280 | 0.38 | 0.12 |
| Margaric acid (17:0) at 8y | 408 | 0.46 | 0.055 | 279 | 0.47 | 0.055 |
| Margaric acid (17:0) at 16y | 406 | 0.45 | 0.051 | 280 | 0.46 | 0.046 |
| Stearic acid (18:0) at 8y | 408 | 16.73 | 0.99 | 279 | 16.57 | 1.05 |
| Stearic acid (18:0) at 16y | 406 | 14.86 | 1.25 | 280 | 14.98 | 0.91 |
| Oleic acid (18:1) at 8y | 408 | 15.3 | 1.43 | 279 | 15.27 | 1.85 |
| Oleic acid (18:1) at 16y | 406 | 12.96 | 1.17 | 280 | 13.17 | 1.23 |
| Linoleic acid (LA, 18:2) at 8y | 408 | 21.36 | 1.82 | 279 | 21.21 | 2.04 |
| Linoleic acid (LA, 18:2) at 16y | 406 | 21.99 | 2.04 | 280 | 21.93 | 2.11 |
| γ-Linolenic acid (18:3) at 8y | 380 | 0.072 | 0.039 | 256 | 0.076 | 0.045 |
| γ-Linolenic acid (18:3) at 16y | 406 | 0.074 | 0.034 | 280 | 0.085 | 0.04 |
| α-Linolenic acid (ALA, 18:3n-3) at 8y | 408 | 0.24 | 0.065 | 278 | 0.25 | 0.073 |
| α-Linolenic acid (ALA, 18:3n-3) at 16y | 406 | 0.30 | 0.087 | 280 | 0.29 | 0.078 |
| Dihomo-γ-linoleic acid (20:3) at 8y | 408 | 2.48 | 0.49 | 279 | 2.51 | 0.51 |
| Dihomo-γ-linoleic acid (20:3) at 16y | 406 | 3.04 | 0.62 | 280 | 3.12 | 0.62 |
| Arachidonic acid (AA, 20:4) at 8y | 408 | 5.55 | 1.35 | 279 | 5.78 | 1.29 |
| Arachidonic acid (AA, 20:4) at 16y | 406 | 8.74 | 1.30 | 280 | 9.23 | 1.24 |
| Eicosapentaeonic acid (EPA, 20:5n-3) at 8y | 408 | 0.66 | 0.24 | 279 | 0.69 | 0.27 |
| Eicosapentaeonic acid (EPA, 20:5n-3) at 16y | 406 | 1.12 | 0.52 | 280 | 1.12 | 0.47 |
| Docosapentaeonic acid (DPA, 22:5n-3) at 8y | 408 | 0.58 | 0.19 | 279 | 0.62 | 0.2 |
| Docosapentaeonic acid (DPA, 22:5n-3) at 16y | 406 | 0.95 | 0.21 | 280 | 1.06 | 0.21 |
| Docosahexaeonic acid (DHA, 22:6n-3) at 8y | 408 | 2.12 | 0.65 | 279 | 2.16 | 0.7 |
| Docosahexaeonic acid (DHA, 22:6n-3) at 16y | 406 | 3.52 | 0.93 | 280 | 3.35 | 0.74 |

| **Supplemental Table 2.** Spearman's rank correlation (p-value) between the proportions of different plasma PUFA at 8 and 16 years of age. | | | | | | | |
| --- | --- | --- | --- | --- | --- | --- | --- |
|  | **ALA 16y** | **∑VLC n-3 8y** | **∑VLC n-3 16y** | **LA 8y** | **LA 16y** | **AA 8y** | **AA 16y** |
| **ALA 8y** | 0.17 (<0.001) | 0.21 (<0.001) |  | 0.34 (<0.001) |  | 0.14 (<0.001) |  |
| **ALA 16y** |  |  | -0.079 (0.038) |  | 0.30 (<0.001) |  | -0.33 (<0.001) |
| **∑VLC n-3 8y** | |  | 0.34 (<0.001) | -0.029 (0.45) |  | 0.81 (<0.001) |  |
| **∑VLC n-3 16y** | |  |  |  | -0.35 (<0.001) |  | 0.30 (<0.001) |
| **LA 8y** |  |  |  |  | 0.25 (<0.001) | 0.038 (0.32) |  |
| **LA 16y** |  |  |  |  |  |  | -0.36 (<0.001) |
| **AA 8y** |  |  |  |  |  |  | 0.21 (<0.001) |
| AA, arachidonic acid; ALA, α-linolenic acid; LA, linoleic acid; PUFA, polyunsaturated fatty acid; ∑VLC n-3 PUFA, sum of very long chain n-3 polyunsaturated fatty acids. | | | | | | | |

| **Supplemental Table 3.** Baseline characteristics of the participants of the present study (n=688) and of the entire BAMSE cohort (n=4089). | | | | | | | |
| --- | --- | --- | --- | --- | --- | --- | --- |
|  |  | **Included** | | | **Whole BAMSE cohort** | | |
|  |  | **n** | **Mean (or %)** | **SD** | **n** | **Mean (or %)** | **SD** |
| **Sex** | | 688 |  |  | 4089 |  |  |
|  | Females | 408 | 59% |  | 2024 | 49% |  |
|  | Males | 280 | 41% |  | 2065 | 51% |  |
| **Parental SES at baseline** | | 683 |  |  | 4018 |  |  |
|  | Professional | 605 | 89% |  | 3323 | 83% |  |
|  | Non-professional | 78 | 11% |  | 695 | 17% |  |
| **Birth weight (g)** | | 689 | 3504 | 559 | 4044 | 3530 | 558 |
| **Parental smoking at baseline** | | 684 |  |  | 4067 |  |  |
|  | No | 556 | 81% |  | 3212 | 79% |  |
|  | Yes | 128 | 19% |  | 855 | 21% |  |
| **Maternal smoking during pregnancy** | | 688 |  |  | 4088 |  |  |
|  | No | 614 | 89% |  | 3561 | 87% |  |
|  | Yes | 73 | 11% |  | 527 | 13% |  |
| ETS, environmental tobacco smoke; SD, standard deviation; SES, socioeconomic status. | | | | | | | |

| **Supplemental Table 4**. Cardiometabolic outcomes at the 24-year follow-up in the participants of this study (n=688) and in all participants taking part in the clinical examination at 24 years (n=2271). | | | | | | | |
| --- | --- | --- | --- | --- | --- | --- | --- |
| **Outcomes at the 24y follow-up** | **Included** | | | | **Whole 24y clinical examination** | | |
|  | **n** | **Mean** | | **SD** | **n** | **Mean** | **SD** |
| **BMI at 24y (kg/m^2^)** | 688 | 23.14 | 3.74 | | 2270 | 23.13 | 3.88 |
| **Waist circumference (cm)** | 686 | 78.7 | 9.7 | | 2256 | 79.3 | 10.3 |
| **Fat mass % (%)** | 675 | 22.5 | 8.0 | | 2234 | 22.3 | 7.9 |
| **Systolic BP (mmHg)** | 688 | 122 | 12 | | 2268 | 122 | 12 |
| **Diastolic BP (mmHg)** | 688 | 76 | 8.1 | | 2268 | 75 | 8.1 |
| **Triglycerides (mmol/L)** | 682 | 1.07 | 0.55 | | 2213 | 1.08 | 0.58 |
| **Total cholesterol (mmol/L)** | 682 | 4.20 | 0.80 | | 2213 | 4.21 | 0.8 |
| **HDL-C (mmol/L)** | 682 | 1.59 | 0.41 | | 2213 | 1.57 | 0.41 |
| **LDL-C (mmol/L)** | 680 | 2.14 | 0.71 | | 2204 | 2.15 | 0.71 |
| BMI, body mass index; BP, blood pressure; HDL-C, high-density lipoprotein cholesterol; LDL-C, low-density lipoprotein cholesterol; SD, standard deviation. | | | | | | | |

| **Supplemental Table 5**. Crude and multivariable-adjusted linear regression models of plasma PUFA proportions at 8 and 16 years and BMI at 24 years. | | | | | |
| --- | --- | --- | --- | --- | --- |
|  |  | **All** | | | |
|  |  | **n** | **B** | **95% CI** | **p-value** |
| **ALA 8y** | |  |  |  |  |
|  | Crude | 686 | -0.81 | -4.9; 3.3 | 0.70 |
|  | Adjusted | 671 | -1.0 | -5.2; 3.1 | 0.62 |
| **ALA 16y** | |  |  |  |  |
|  | Crude | 686 | -1.6 | -5.0; 1.7 | 0.35 |
|  | Adjusted | 657 | -1.7 | -5.2; 1.8 | 0.34 |
| **∑VLC n-3 8y** | |  |  |  |  |
|  | Crude | 687 | -0.086 | -0.36; 0.19 | 0.54 |
|  | Adjusted | 673 | -0.029 | -0.31; 0.25 | 0.84 |
| **∑VLC n-3 16y** | |  |  |  |  |
|  | Crude | 686 | -0.10 | -0.31; 0.11 | 0.34 |
|  | Adjusted | 657 | 0.021 | -0.20; 0.24 | 0.85 |
| **LA 8y** | |  |  |  |  |
|  | Crude | 687 | -0.24 | -0.38; -0.090 | 0.002 |
|  | Adjusted | 673 | -0.22 | -0.37; -0.075 | 0.003 |
| **LA 16y** | |  |  |  |  |
|  | Crude | 686 | -0.23 | -0.36; -0.090 | 0.001 |
|  | Adjusted | 657 | -0.23 | -0.36; -0.087 | 0.002 |
| **AA 8y** | |  |  |  |  |
|  | Crude | 687 | 0.030 | -0.18; 0.24 | 0.78 |
|  | Adjusted | 673 | 0.053 | -0.16; 0.27 | 0.63 |
| **AA 16y** | |  |  |  |  |
|  | Crude | 686 | 0.15 | -0.068; 0.37 | 0.18 |
|  | Adjusted | 657 | 0.14 | -0.088; 0.36 | 0.23 |
| Crude model: unadjusted | | |  |  |  |
| Adjusted model: adjusted for sex, parental socioeconomic status at baseline (professional or non-professional worker), occupation at 24y (studying, employed, other), education at 24y (studies after secondary school or no), smoking at 24y (yes or no), snus at 24y (yes or no), sedentary level at 24y (≤6, 7-9, or ≥10 hours), birth weight (g), parental smoking at baseline (yes or no), maternal smoking during pregnancy (yes or no), dietary fiber intake at 8y (g, in models of plasma PUFA at 8y) or dietary fiber intake at 16y (g, in models of plasma PUFA at 16y). AA, arachidonic acid; ALA, α-linolenic acid; BMI, body mass index; CI, confidence interval; LA, linoleic acid; PUFA, polyunsaturated fatty acid; ∑VLC n-3 PUFA, sum of very long chain n-3 polyunsaturated fatty acids. | | | | | |

| **Supplemental Table 6**. Sensitivity analysis of multivariable-adjusted linear regression models of plasma PUFA proportions at 8 and 16 years and BMI, waist circumference and fat mass % at 24 years, additionally adjusted for total energy intake^1^. | | | | | |
| --- | --- | --- | --- | --- | --- |
| BMI | | | | | |
|  |  | Females | | Males | |
|  |  | n | B (95% CI) | n | B (95% CI) |
| ALA |  |  |  |  |  |
|  | 8y | 399 | -1.0 (-7.0, 4.9) | 273 | 0.067 (-5.7, 5.8) |
|  | 16y | 388 | -6.2 (-11, -1.7) | 269 | 4.3 (-1.3, 9.8) |
| ∑VLC n-3 | |  |  |  |  |
|  | 8y | 399 | 0.18 (-0.21, 0.58) | 274 | -0.22 (-0.63, 0.19) |
|  | 16y | 388 | 0.043 (-0.24, 0.32) | 269 | 0.034 (-0.34, 0.41) |
| LA |  |  |  |  |  |
|  | 8y | 399 | -0.41 (-0.62, -0.21) | 274 | -0.037 (-0.24, 0.17) |
|  | 16y | 388 | -0.36 (-0.55, -0.17) | 269 | -0.098 (-0.30, 0.11) |
| AA |  |  |  |  |  |
|  | 8y | 399 | 0.14 (-0.15, 0.42) | 274 | -0.029 (-0.36, 0.31) |
|  | 16y | 388 | 0.17 (-0.13, 0.47) | 269 | 0.19 (-0.16, 0.54) |
| Waist circumference | | | | | |
|  |  | Females | | Males | |
|  |  | n | B (95% CI) | n | B (95% CI) |
| ALA | |  |  |  |  |
|  | 8y | 399 | -7.3 (-21, 5.9) | 271 | -1.2 (-16, 14) |
|  | 16y | 388 | -15 (-25, -4.5) | 267 | 11 (-3.4, 25) |
| ∑VLC n-3 | |  |  |  |  |
|  | 8y | 399 | 0.071 (-0.82, 0.96) | 272 | -0.64 (-1.7, 0.43) |
|  | 16y | 388 | 0.21 (-0.41, 0.84) | 267 | -0.16 (-1.1, 0.80) |
| LA |  |  |  |  |  |
|  | 8y | 399 | -0.88 (-1.34, -0.41) | 272 | 0.060 (-0.48, 0.60) |
|  | 16y | 388 | -0.95 (-1.37, -0.54) | 267 | -0.31 (-0.84, 0.22) |
| AA |  |  |  |  |  |
|  | 8y | 399 | -0.065 (-0.71, 0.58) | 272 | -0.33 (-1.2, 0.54) |
|  | 16y | 388 | 0.25 (-0.42, 0.92) | 267 | 0.16 (-0.75, 1.1) |
| Fat mass % | | | | | |
|  |  | Females | | Males | |
|  |  | n | B (95% CI) | n | B (95% CI) |
| ALA | |  |  |  |  |
|  | 8y | 391 | -3.6 (-13, 5.8) | 268 | -0.43 (-11, 9.8) |
|  | 16y | 380 | -10 (-17, -3.1) | 264 | 3.3 (-6.5, 13) |
| ∑VLC n-3 | |  |  |  |  |
|  | 8y | 391 | 0.25 (-0.39, 0.88) | 269 | -0.52 (-1.3, 0.21) |
|  | 16y | 380 | 0.13 (-0.31, 0.57) | 264 | -0.23 (-0.88, 0.43) |
| LA |  |  |  |  |  |
|  | 8y | 391 | -0.65 (-0.98, -0.32) | 269 | -0.17 (-0.54, 0.20) |
|  | 16y | 380 | -0.55 (-0.85, -0.26) | 264 | -0.22 (-0.59, 0.14) |
| AA |  |  |  |  |  |
|  | 8y | 391 | 0.076 (-0.38, 0.53) | 269 | -0.088 (-0.70, 0.52) |
|  | 16y | 380 | 0.14 (-0.33, 0.61) | 264 | 0.33 (-0.29, 0.95) |
| ^1^Models adjusted for parental socioeconomic status at baseline (professional or non-professional worker), occupation at 24y (studying, employed, other), education at 24y (studies after secondary school or no), smoking at 24y (yes or no), snus at 24y (yes or no), sedentary level at 24y (≤6, 7-9, or ≥10 hours), birth weight (g), parental smoking at baseline (yes or no), maternal smoking during pregnancy (yes or no), dietary fiber intake at 8y (g, in models of plasma PUFA at 8y) or dietary fiber intake at 16y (g, in models of plasma PUFA at 16y), total energy intake at 8y (kcal, in models of plasma PUFA at 8y) or total energy intake at 16y (kcal, in models of plasma PUFA at 16y). AA, arachidonic acid ALA, α-linolenic acid; BMI, body mass index; CI, confidence interval; LA, linoleic acid; PUFA, polyunsaturated fatty acid; ∑VLC n-3 PUFA, sum of very long chain n-3 polyunsaturated fatty acids. | | | | | |

| **Supplemental Table 7**. Sensitivity analysis of multivariable-adjusted linear regression models of plasma PUFA proportions at 8 and 16 years and BMI, waist circumference and fat mass % at 24 years, additionally adjusted for proportions of palmitic acid (16:0), stearic acid (18:0), and oleic acid (18:1n-9)^1^. | | | | | |
| --- | --- | --- | --- | --- | --- |
| BMI | | | | | |
|  |  | Females | | Males | |
|  |  | n | B (95% CI) | n | B (95% CI) |
| ALA |  |  |  |  |  |
|  | 8y | 399 | -0.27 (-7.0, 6.4) | 273 | -0.048 (-7.1, 7.1) |
|  | 16y | 388 | -5.3 (-10, -0.65) | 269 | 6.0 (0.37, 12) |
| ∑VLC n-3 | |  |  |  |  |
|  | 8y | 399 | 0.62 (0.068, 1.2) | 274 | -0.27 (-0.81, 0.28) |
|  | 16y | 388 | 0.061 (-0.27, 0.40) | 269 | -0.12 (-0.53, 0.28) |
| LA |  |  |  |  |  |
|  | 8y | 399 | -0.48 (-0.73, -0.23) | 274 | -0.014 (-0.29, 0.26) |
|  | 16y | 388 | -0.30 (-0.51, -0.082) | 269 | -0.14 (-0.38, 0.094) |
| AA |  |  |  |  |  |
|  | 8y | 399 | 0.48 (0.086, 0.87) | 274 | 0.15 (-0.30, 0.60) |
|  | 16y | 388 | 0.39 (0.053, 0.73) | 269 | 0.11 (-0.28, 0.51) |
| Waist circumference | | | | | |
|  |  | Females | | Males | |
|  |  | n | B (95% CI) | n | B (95% CI) |
| ALA |  |  |  |  |  |
|  | 8y | 399 | -5.4 (-20, 9.6) | 271 | -0.62 (-19, 18) |
|  | 16y | 388 | -11 (-21, -1.0) | 267 | 17 (2.9, 32) |
| ∑VLC n-3 | |  |  |  |  |
|  | 8y | 399 | 1.14 (-0.095, 2.4) | 272 | -0.71 (-2.1, 0.73) |
|  | 16y | 388 | 0.33 (-0.40, 1.1) | 267 | -0.41 (-1.4, 0.64) |
| LA |  |  |  |  |  |
|  | 8y | 399 | -0.87 (-1.43, -0.30) | 272 | 0.29 (-0.42, 1.0) |
|  | 16y | 388 | -0.71 (-1.2, -0.25) | 267 | -0.18 (-0.79, 0.43) |
| AA |  |  |  |  |  |
|  | 8y | 399 | 0.57 (-0.32, 1.4) | 272 | -0.21 (-1.4, 0.98) |
|  | 16y | 388 | 0.81 (0.073, 1.5) | 267 | 0.27 (-0.75, 1.3) |
| Fat mass % | | | | | |
|  |  | Females | | Males | |
|  |  | n | B (95% CI) | n | B (95% CI) |
| ALA |  |  |  |  |  |
|  | 8y | 391 | -2.1 (-13, 8.5) | 268 | -0.62 (-13, 12) |
|  | 16y | 380 | -9.1 (-17, -1.7) | 264 | 5.6 (-4.4, 16) |
| ∑VLC n-3 | |  |  |  |  |
|  | 8y | 391 | 1.1 (0.22, 2.0) | 269 | -0.41 (-1.4, 0.57) |
|  | 16y | 380 | 0.24 (-0.30, 0.77) | 264 | -0.34 (-1.1, 0.38) |
| LA |  |  |  |  |  |
|  | 8y | 391 | -0.70 (-1.1, -0.31) | 269 | -0.067 (-0.56, 0.43) |
|  | 16y | 380 | -0.48 (-0.82, -0.14) | 264 | -0.21 (-0.63, 0.20) |
| AA |  |  |  |  |  |
|  | 8y | 391 | 0.055 (-0.071, 1.2) | 269 | 0.43 (-0.38, 1.2) |
|  | 16y | 380 | 0.45 (-0.082, 0.98) | 264 | 0.41 (-0.29, 1.1) |
| ^1^Models adjusted for parental socioeconomic status at baseline (professional or non-professional worker), occupation at 24y (studying, employed, other), education at 24y (studies after secondary school or no), smoking at 24y (yes or no), snus at 24y (yes or no), sedentary level at 24y (≤6, 7-9, or ≥10 hours), birth weight (g), parental smoking at baseline (yes or no), maternal smoking during pregnancy (yes or no), dietary fiber intake at 8y (g, in models of plasma PUFA at 8y) or dietary fiber intake at 16y (g, in models of plasma PUFA at 16y), total energy intake at 8y (kcal, in models of plasma PUFA at 8y) or total energy intake at 16y (kcal, in models of plasma PUFA at 16y), and plasma phospholipid proportions of palmitic, stearic and oleic acids at 8y (in models of plasma PUFA at 8y) or at 16 years (in models of plasma PUFA at 16y). AA, arachidonic acid ALA, α-linolenic acid; BMI, body mass index; CI, confidence interval; LA, linoleic acid; PUFA, polyunsaturated fatty acid; ∑VLC n-3 PUFA, sum of very long chain n-3 polyunsaturated fatty acids. | | | | | |

| **Supplemental Table 8.** Sensitivity analysis of multivariable-adjusted linear regression models of plasma PUFA proportions at 8 and 16 years and systolic and diastolic blood pressure at 24 years, additionally adjusted for total energy intake^1^. | | | | | |
| --- | --- | --- | --- | --- | --- |
| Systolic blood pressure | | | | | |
|  |  | Females | | Males | |
|  |  | n | B (95% CI) | n | B (95% CI) |
| ALA | |  |  |  |  |
|  | 8y | 399 | -7.6 (-21, 5.9) | 273 | 2.1 (-17, 21) |
|  | 16y | 388 | 3.0 (-7.4, 13) | 269 | 0.32 (-18, 18) |
| ∑VLC n-3 | |  |  |  |  |
|  | 8y | 399 | -0.15 (-1.1, 0.75) | 274 | 0.11 (-1.2, 1.4) |
|  | 16y | 388 | 0.45 (-0.19, 1.1) | 269 | -0.39 (-1.6, 0.81) |
| LA |  |  |  |  |  |
|  | 8y | 399 | -0.50 (-0.98, -0.026) | 274 | 0.078 (-0.60, 0.75) |
|  | 16y | 388 | -0.41 (-0.84, 0.026) | 269 | 0.34 (-0.33, 1.0) |
| AA |  |  |  |  |  |
|  | 8y | 399 | -0.21 (0.86, 0.45) | 274 | 0.037 (-1.1, 1.1) |
|  | 16y | 388 | -0.16 (0.85, 0.52) | 269 | -0.10 (-1.2, 1.0) |
| Diastolic blood pressure | | | | | |
|  |  | Females | | Males | |
|  |  | n | B (95% CI) | n | B (95% CI) |
| ALA | |  |  |  |  |
|  | 8y | 399 | -4.5 (-16, 7.2) | 273 | -3.0 (-18, 12) |
|  | 16y | 388 | -2.1 (-11, 7.0) | 269 | -12 (-26, 1.6) |
| ∑VLC n-3 | |  |  |  |  |
|  | 8y | 399 | -0.52 (-1.3, 0.26) | 274 | 0.26 (-0.78, 1.3) |
|  | 16y | 388 | -0.19 (-0.37, 0.75) | 269 | 0.17 (-0.77, 1.1) |
| LA |  |  |  |  |  |
|  | 8y | 399 | -0.48 (-0.89, -0.063) | 274 | -0.24 (-0.76, 0.29) |
|  | 16y | 388 | -0.40 (-0.78, -0.027) | 269 | -0.25 (-0.77, 0.27) |
| AA |  |  |  |  |  |
|  | 8y | 399 | -0.33 (0.90, 0.24) | 274 | 0.13 (-0.72, 0.97) |
|  | 16y | 388 | 0.13 (-0.46, 0.73) | 269 | 0.53 (-0.35, 1.4) |
| ^1^Models adjusted for parental socioeconomic status at baseline (professional or non-professional worker), occupation at 24y (studying, employed, other), education at 24y (studies after secondary school or no), smoking at 24y (yes or no), snus at 24y (yes or no), sedentary level at 24y (≤6, 7-9, or ≥10 hours), birth weight (g), parental smoking at baseline (yes or no), maternal smoking during pregnancy (yes or no), dietary fiber intake at 8y (g, in models of plasma PUFA at 8y) or dietary fiber intake at 16y (g, in models of plasma PUFA at 16y), total energy intake at 8y (kcal, in models of plasma PUFA at 8y) or total energy intake at 16y (kcal, in models of plasma PUFA at 16y). AA, arachidonic acid ALA, α-linolenic acid; CI, confidence interval; LA, linoleic acid; PUFA, polyunsaturated fatty acid; ∑VLC n-3 PUFA, sum of very long chain n-3 polyunsaturated fatty acids. | | | | | |

| **Supplemental Table 9.** Sensitivity analysis of multivariable-adjusted linear regression models of plasma PUFA proportions at 8 and 16 years and blood pressure at 24 years, additionally adjusted for proportions of palmitic acid (16:0), stearic acid (18:0), and oleic acid (18:1n-9)^1^. | | | | | |
| --- | --- | --- | --- | --- | --- |
| Systolic blood pressure | | | | | |
|  |  | Females | | Males | |
|  |  | n | B (95% CI) | n | B (95% CI) |
| ALA |  |  |  |  |  |
|  | 8y | 399 | -4.4 (-20, 11) | 273 | -4.9 (-28, 18) |
|  | 16y | 388 | 5.2 (-5.5, 16) | 269 | 1.2 (-18, 20) |
| ∑VLC n-3 | |  |  |  |  |
|  | 8y | 399 | 0.77 (-0.48, 2.0) | 274 | -0.24 (-2.0, 1.6) |
|  | 16y | 388 | 0.56 (-0.20, 1.3) | 269 | -0.87 (-2.2, 0.46) |
| LA |  |  |  |  |  |
|  | 8y | 399 | -0.39 (-0.97, 0.18) | 274 | 0.056 (-0.84, 0.95) |
|  | 16y | 388 | -0.29 (-0.78, 0.20) | 269 | 0.26 (-0.52, 1.0) |
| AA |  |  |  |  |  |
|  | 8y | 399 | 0.37 (-0.52, 1.3) | 274 | 0.053 (-1.4, 1.5) |
|  | 16y | 388 | -0.020 (-0.79, 0.75) | 269 | -0.55 (-1.9, 0.75) |
| Diastolic blood pressure | | | | | |
|  |  | Females | | Males | |
|  |  | n | B (95% CI) | n | B (95% CI) |
| ALA |  |  |  |  |  |
|  | 8y | 399 | -4.0 (-17, 9.0) | 273 | -7.0 (-25, 11) |
|  | 16y | 388 | -1.4 (-11, 8.0) | 269 | -12 (-27, 2.3) |
| ∑VLC n-3 | |  |  |  |  |
|  | 8y | 399 | 0.24 (-0.84, 1.32) | 274 | 0.50 (-0.89, 1.9) |
|  | 16y | 388 | 0.48 (-0.19, 1.1) | 269 | 0.014 (-1.0, 1.1) |
| LA |  |  |  |  |  |
|  | 8y | 399 | -0.26 (-0.75, 0.23) | 274 | -0.33 (-1.0, 0.36) |
|  | 16y | 388 | -0.36 (-0.79, 0.073) | 269 | -0.38 (-0.98, 0.22) |
| AA |  |  |  |  |  |
|  | 8y | 399 | 0.50 (-0.27, 1.3) | 274 | 0.41 (-0.73, 1.6) |
|  | 16y | 388 | 0.45 (-0.22, 1.1) | 269 | 0.53 (-0.48, 1.5) |
| ^1^Models adjusted for parental socioeconomic status at baseline (professional or non-professional worker), occupation at 24y (studying, employed, other), education at 24y (studies after secondary school or no), smoking at 24y (yes or no), snus at 24y (yes or no), sedentary level at 24y (≤6, 7-9, or ≥10 hours), birth weight (g), parental smoking at baseline (yes or no), maternal smoking during pregnancy (yes or no), dietary fiber intake at 8y (g, in models of plasma PUFA at 8y) or dietary fiber intake at 16y (g, in models of plasma PUFA at 16y), total energy intake at 8y (kcal, in models of plasma PUFA at 8y) or total energy intake at 16y (kcal, in models of plasma PUFA at 16y), and plasma phospholipid proportions of palmitic, stearic and oleic acids at 8y (in models of plasma PUFA at 8y) or at 16 years (in models of plasma PUFA at 16y). AA, arachidonic acid ALA, α-linolenic acid; CI, confidence interval; LA, linoleic acid; PUFA, polyunsaturated fatty acid; ∑VLC n-3 PUFA, sum of very long chain n-3 polyunsaturated fatty acids. | | | | | |

| **Supplemental Table 10.** Sensitivity analysis of multivariable-adjusted linear regression models of plasma PUFA proportions at 8 and 16 years and blood lipids at 24 years, additionally adjusted for total energy intake^1^. | | | | | |
| --- | --- | --- | --- | --- | --- |
| Triglycerides | | | | | |
|  |  | Females | | Males | |
|  |  | n | B (95% CI) | n | B (95% CI) |
| ALA | |  |  |  |  |
|  | 8y | 393 | 0.54 (-0.14, 1.2) | 273 | -0.76 (-1.8, 0.33) |
|  | 16y | 382 | -0.25 (-0.78, 0.27) | 269 | 0.65 (-0.39, 1.7) |
| ∑VLC n-3 | |  |  |  |  |
|  | 8y | 393 | -0.0038 (-0.049, 0.042) | 274 | -0.0053 (-0.083, 0.072) |
|  | 16y | 382 | 0.023 (-0.0091, 0.056) | 269 | -0.037 (-0.11, 0.033) |
| LA |  |  |  |  |  |
|  | 8y | 393 | 0.0019 (-0.023, 0.026) | 274 | -0.0031 (-0.042, 0.036) |
|  | 16y | 382 | -0.025 (-0.047, -0.0034) | 269 | 0.014 (-0.025, 0.053) |
| AA |  |  |  |  |  |
|  | 8y | 393 | -0.0057 (-0.039, 0.027) | 274 | -0.016 (-0.080, 0.047) |
|  | 16y | 382 | -0.0030 (-0.038, 0.032) | 269 | -0.028 (-0.094, 0.038) |
| Total cholesterol | | | | | |
|  |  | Females | | Males | |
|  |  | n | B (95% CI) | n | B (95% CI) |
| ALA |  |  |  |  |  |
|  | 8y | 393 | 0.61 (-0.64, 1.9) | 273 | 0.22 (-1.0, 1.5) |
|  | 16y | 382 | -0.55 (-1.5, 0.42) | 269 | -1.0 (-2.2, 0.19) |
| ∑VLC n-3 | |  |  |  |  |
|  | 8y | 393 | -0.036 (-0.12, 0.048) | 274 | 0.0079 (-0.081, 0.097) |
|  | 16y | 382 | 0.013 (-0.047, 0.073) | 269 | -0.018 (-0.10, 0.063) |
| LA |  |  |  |  |  |
|  | 8y | 393 | -0.018 (-0.063, 0.027) | 274 | -0.029 (-0.074, 0.015) |
|  | 16y | 382 | -0.041 (-0.081, -0.00015) | 269 | -0.0029 (-0.048, 0.042) |
| AA |  |  |  |  |  |
|  | 8y | 393 | -0.048 (-0.11, 0.012) | 274 | 0.011 (-0.062, 0.083) |
|  | 16y | 382 | -0.052 (-0.12, 0.012) | 269 | -0.0062 (-0.083, 0.071) |
| HDL-C | | | | | |
|  |  | Females | | Males | |
|  |  | n | B (95% CI) | n | B (95% CI) |
| ALA |  |  |  |  |  |
|  | 8y | 393 | 0.48 (-0.14, 1.1) | 273 | 0.026 (-0.50, 0.56) |
|  | 16y | 382 | 0.41 (-0.082, 0.90) | 269 | -0.29 (-0.81, 0.22) |
| ∑VLC n-3 | |  |  |  |  |
|  | 8y | 393 | -0.018 (-0.060, 0.024) | 274 | 0.0098 (-0.028, 0.047) |
|  | 16y | 382 | 0.0062 (-0.024, 0.036) | 269 | 0.011 (-0.023, 0.045) |
| LA |  |  |  |  |  |
|  | 8y | 393 | 0.021 (-0.0019, 0.043) | 274 | 0.00050 (-0.019, 0.020) |
|  | 16y | 382 | 0.018 (-0.0022, 0.039) | 269 | 0.0031 (-0.016, 0.022) |
| AA |  |  |  |  |  |
|  | 8y | 393 | -0.016 (-0.046, 0.014) | 274 | -0.0028 (-0.034, 0.028) |
|  | 16y | 382 | -0.0037 (-0.036, 0.029) | 269 | -0.0013 (-0.034, 0.031) |
| LDL-C | | | | | |
|  |  | Females | | Males | |
|  |  | n | B (95% CI) | n | B (95% CI) |
| ALA |  |  |  |  |  |
|  | 8y | 392 | -0.13 (-1.2, 0.94) | 272 | 0.32 (-0.85, 1.5) |
|  | 16y | 381 | -0.89 (-1.7, -0.060) | 268 | -1.1 (-2.2, 0.063) |
| ∑VLC n-3 | |  |  |  |  |
|  | 8y | 392 | -0.025 (-0.096, 0.047) | 273 | 0.0012 (-0.082, 0.084) |
|  | 16y | 381 | -0.011 (-0.062, 0.041) | 268 | -0.031 (-0.11, 0.044) |
| LA |  |  |  |  |  |
|  | 8y | 392 | -0.031 (-0.069, 0.0082) | 273 | -0.028 (-0.070, 0.014) |
|  | 16y | 381 | -0.045 (-0.079, -0.0099) | 268 | -0.0027 (-0.045, 0.039) |
| AA |  |  |  |  |  |
|  | 8y | 392 | -0.029 (-0.081, 0.023) | 273 | 0.015 (-0.052, 0.083) |
|  | 16y | 381 | -0.049 (-0.10, 0.0057) | 268 | -0.0018 (-0.073, 0.069) |
| ^1^Models adjusted for parental socioeconomic status at baseline (professional or non-professional worker), occupation at 24y (studying, employed, other), education at 24y (studies after secondary school or no), smoking at 24y (yes or no), snus at 24y (yes or no), sedentary level at 24y (≤6, 7-9, or ≥10 hours), birth weight (g), parental smoking at baseline (yes or no), maternal smoking during pregnancy (yes or no), dietary fiber intake at 8y (g, in models of plasma PUFA at 8y) or dietary fiber intake at 16y (g, in models of plasma PUFA at 16y). AA, arachidonic acid ALA, α-linolenic acid; CI, confidence interval; HDL-C, high-density lipoprotein cholesterol; LA, linoleic acid; LDL-C, low-density lipoprotein cholesterol; PUFA, polyunsaturated fatty acid; ∑VLC n-3 PUFA, sum of very long chain n-3 polyunsaturated fatty acids. | | | | | |

| **Supplemental Table 11**. Sensitivity analysis of multivariable-adjusted linear regression models of plasma PUFA proportions at 8 and 16 years and blood lipids at 24 years, additionally adjusted for proportions of palmitic acid (16:0), stearic acid (18:0), and oleic acid (18:1n-9)^1^. | | | | | |
| --- | --- | --- | --- | --- | --- |
| Triglycerides | | | | | |
|  |  | Females | | Males | |
|  |  | n | B (95% CI) | n | B (95% CI) |
| ALA | |  |  |  |  |
|  | 8y | 393 | 0.73 (-0.035, 1.5) | 273 | -0.69 (-2.0, 0.65) |
|  | 16y | 382 | -0.24 (-0.78, 0.30) | 269 | 0.78 (-0.30, 1.9) |
| ∑VLC n-3 | | |  |  |  |
|  | 8y | 393 | -0.0066 (-0.071, 0.058) | 274 | 0.018 (-0.086, 0.12) |
|  | 16y | 382 | 0.037 (-0.0016, 0.075) | 269 | -0.042 (-0.12, 0.034) |
| LA |  |  |  |  |  |
|  | 8y | 393 | 0.0028 (-0.027, 0.032) | 274 | -0.00057 (-0.053, 0.052) |
|  | 16y | 382 | 0.024 (-0.048, 0.0011) | 269 | 0.024 (-0.020, 0.069) |
| AA |  |  |  |  |  |
|  | 8y | 393 | -0.014 (-0.059, 0.031) | 274 | -0.023 (-0.11, 0.062) |
|  | 16y | 382 | 0.011 (-0.028, 0.050) | 269 | -0.033 (-0.11, 0.042) |
| Total cholesterol | | | | | |
|  |  | Females | | Males | |
|  |  | n | B (95% CI) | n | B (95% CI) |
| ALA | |  |  |  |  |
|  | 8y | 393 | 1.5 (0.12, 2.9) | 273 | -0.067 (-1.6, 1.5) |
|  | 16y | 382 | -0.16 (-1.1, 0.82) | 269 | -0.75 (-2.0, 0.50) |
| ∑VLC n-3 | | |  |  |  |
|  | 8y | 393 | 0.039 (-0.080, 0.16) | 274 | 0.050 (-0.068, 0.17) |
|  | 16y | 382 | 0.027 (-0.042, 0.097) | 269 | -0.030 (-0.12, 0.059) |
| LA |  |  |  |  |  |
|  | 8y | 393 | 0.0041 (-0.050, 0.058) | 274 | -0.032 (-0.091, 0.027) |
|  | 16y | 382 | -0.0024 (-0.047, 0.043) | 269 | 0.0086 (-0.043, 0.060) |
| AA |  |  |  |  |  |
|  | 8y | 393 | -0.030 (-0.11, 0.053) | 274 | 0.090 (-0.0075, 0.19) |
|  | 16y | 382 | -0.020 (-0.091, 0.051) | 269 | -0.0063 (-0.093, 0.081) |
| HDL-C | | | | | |
|  |  | Females | | Males | |
|  |  | n | B (95% CI) | n | B (95% CI) |
| ALA | |  |  |  |  |
|  | 8y | 393 | 0.70 (-0.0091, 1.4) | 273 | -0.24 (-0.89, 0.41) |
|  | 16y | 382 | 0.53 (0.033, 1.0) | 269 | -0.33 (-0.86, 0.21) |
| ∑VLC n-3 | | |  |  |  |
|  | 8y | 393 | -0.030 (-0.089, 0.030) | 274 | 0.011 (-0.039, 0.062) |
|  | 16y | 382 | 0.0068 (-0.042, 0.029) | 269 | 0.010 (-0.028, 0.048) |
| LA |  |  |  |  |  |
|  | 8y | 393 | 0.029 (0.0017, 0.055) | 274 | 0.0016 (-0.024, 0.027) |
|  | 16y | 382 | 0.020 (-0.0027, 0.043) | 269 | 0.0027 (-0.019, 0.025) |
| AA |  |  |  |  |  |
|  | 8y | 393 | -0.038 (-0.079, 0.0037) | 274 | -0.00058 (-0.042, 0.041) |
|  | 16y | 382 | -0.028 (-0.064, 0.0075) | 269 | -0.0046 (-0.042, 0.032) |
| LDL-C | | | | | |
|  |  | Females | | Males | |
|  |  | n | B (95% CI) | n | B (95% CI) |
| ALA | |  |  |  |  |
|  | 8y | 392 | 0.44 (-0.76, 1.6) | 272 | 0.21 (-1.2, 1.6) |
|  | 16y | 381 | -0.60 (-1.4, 0.22) | 268 | -0.84 (-2.0, 0.32) |
| ∑VLC n-3 | | |  |  |  |
|  | 8y | 392 | 0.049 (-0.053, 0.15) | 273 | 0.036 (-0.074, 0.15) |
|  | 16y | 381 | -0.011 (-0.047, 0.070) | 268 | -0.040 (-0.12, 0.043) |
| LA |  |  |  |  |  |
|  | 8y | 392 | -0.016 (-0.062, 0.030) | 273 | -0.030 (-0.085, 0.025) |
|  | 16y | 381 | -0.0077 (-0.046, 0.030) | 268 | 0.0077 (-0.041, 0.056) |
| AA |  |  |  |  |  |
|  | 8y | 392 | 0.0089 (-0.061, 0.079) | 273 | 0.093 (0.0027, 0.18) |
|  | 16y | 381 | 0.00070 (-0.059, 0.060) | 268 | 0.0037 (-0.077, 0.085) |
| ^1^Models adjusted for parental socioeconomic status at baseline (professional or non-professional worker), occupation at 24y (studying, employed, other), education at 24y (studies after secondary school or no), smoking at 24y (yes or no), snus at 24y (yes or no), sedentary level at 24y (≤6, 7-9, or ≥10 hours), birth weight (g), parental smoking at baseline (yes or no), maternal smoking during pregnancy (yes or no), dietary fiber intake at 8y (g, in models of plasma PUFA at 8y) or dietary fiber intake at 16y (g, in models of plasma PUFA at 16y), and plasma phospholipid proportions of palmitic, stearic and oleic acids at 8y (in models of plasma PUFA at 8y) or at 16 years (in models of plasma PUFA at 16y). AA, arachidonic acid ALA, α-linolenic acid; CI, confidence interval; HDL-C, high-density lipoprotein cholesterol; LA, linoleic acid; LDL-C, low-density lipoprotein cholesterol; PUFA, polyunsaturated fatty acid; ∑VLC n-3 PUFA, sum of very long chain n-3 polyunsaturated fatty acids. | | | | | |

**
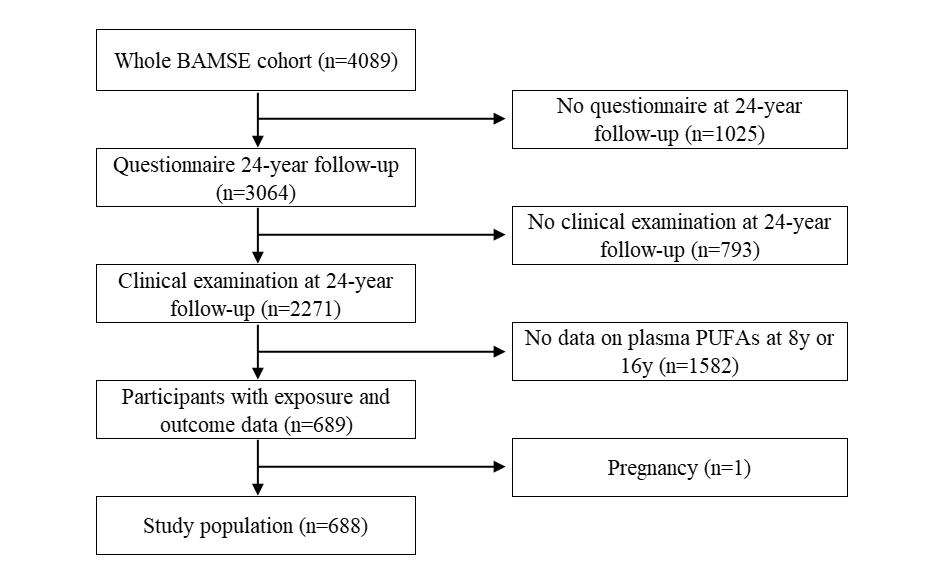
**

**Supplemental Figure 1.** Flowchart of study participants.


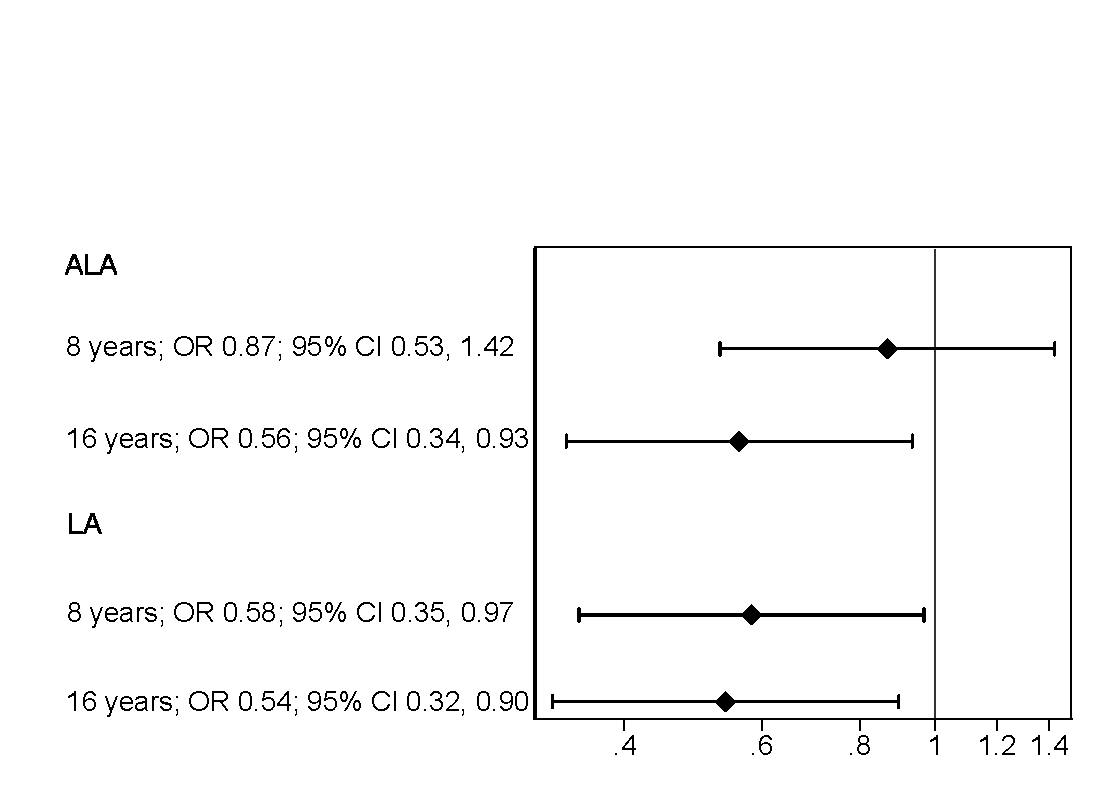


**Supplemental Figure 2.** Associations between plasma proportions of ALA and LA (high compared with low) at 8 and 16 years and overweight or obesity at 24 years in females. Group below the median as reference. Median plasma proportions of the PUFA were as following: ALA 8y 0.23%, ALA 16y 0.28%, LA 8y 21.36%, LA 16y 21.86%.

Logistic regression models adjusted for socioeconomic status at baseline, occupation at 24y, education at 24y, smoking at 24y, snus at 24y, sedentary level at 24y, birth weight, parental smoking at baseline, maternal smoking during pregnancy, dietary fiber intake at 8y (in models of plasma PUFA at 8y) or dietary fiber intake at 16y (in models of plasma PUFA at 16y).

Abbreviations: ALA, α-linolenic acid; CI, confidence interval; LA, linoleic acid; OR, odds ratio; PUFA, polyunsaturated fatty acid.
